# Heme oxygenase-1—Dependent anti-inflammatory effects of atorvastatin in zymosan-injected subcutaneous air pouch in mice

**DOI:** 10.1371/journal.pone.0216405

**Published:** 2019-05-09

**Authors:** Ghewa A. El-Achkar, May F. Mrad, Charbel A. Mouawad, Bassam Badran, Ayad A. Jaffa, Roberto Motterlini, Eva Hamade, Aida Habib

**Affiliations:** 1 Department of Biochemistry and Molecular Genetics, American University of Beirut, Beirut, Lebanon; 2 INSERM U955, Equipe 12, University Paris-Est, Faculty of Medicine, Créteil, France; 3 Nehme and Therese Tohme Multiple Sclerosis Center, American University of Beirut Medical Center, Beirut, Lebanon; 4 Laboratory of Cancer Biology and Molecular Immunology, Faculty of Sciences I, Lebanese University, Hadath, Beirut, Lebanon; 5 INSERM-U1149, CNRS-ERL8252, Centre de Recherche sur l’Inflammation, Sorbonne Paris Cité, Laboratoire d’Excellence Inflamex, Faculté de Médecine, Site Xavier Bichat, Université de Paris, Paris, France; University of Missouri Health Care, UNITED STATES

## Abstract

Statins exert pleiotropic and beneficial anti-inflammatory and antioxidant effects. We have previously reported that macrophages treated with statins increased the expression of heme oxygenase-1 (HO-1), an inducible anti-inflammatory and cytoprotective stress protein, responsible for the degradation of heme. In the present study, we investigated the effects of atorvastatin on inflammation in mice and analyzed its mechanism of action *in vivo*. Air pouches were established in 8 week-old female C57BL/6J mice. Atorvastatin (5 mg/kg, i.p.) and/or tin protoporphyrin IX (SnPPIX), a heme oxygenase inhibitor (12 mg/kg, i.p.), were administered for 10 days. Zymosan, a cell wall component of Saccharomyces cerevisiae, was injected in the air pouch to trigger inflammation. Cell number and levels of inflammatory markers were determined in exudates collected from the pouch 24 hours post zymosan injection by flow cytometry, ELISA and quantitative PCR. Analysis of the mice treated with atorvastatin alone displayed increased expression of HO-1, arginase-1, C-type lectin domain containing 7A, and mannose receptor C-type 1 in the cells of the exudate of the air pouch. Flow cytometry analysis revealed an increase in monocyte/macrophage cells expressing HO-1 and in leukocytes expressing MRC-1 in response to atorvastatin. Mice treated with atorvastatin showed a significant reduction in cell influx in response to zymosan, and in the expression of proinflammatory cytokines and chemokines such as interleukin-1α, monocyte chemoattractant protein-1 and prostaglandin E_2_. Co-treatment of mice with atorvastatin and tin protoporphyrin IX (SnPPIX), an inhibitor of heme oxygenase, reversed the inhibitory effect of statin on cell influx and proinflammatory markers, suggesting a protective role of HO-1. Flow cytometry analysis of air pouch cell contents revealed prevalence of neutrophils and to a lesser extent of monocytes/macrophages with no significant effect of atorvastatin treatment on the modification of their relative proportion. These findings identify HO-1 as a target for the therapeutic actions of atorvastatin and highlight its potential role as an *in vivo* anti-inflammatory agent.

## Introduction

Statins are competitive inhibitors of 3-hydroxy-3-methylglutaryl coenzyme A (HMG-CoA) reductase and inhibit cholesterol synthesis and low-density lipoprotein cholesterol (LDL-C). Satins have been shown to have many beneficial pleiotropic effects beyond their ability to lower LDL-cholesterol, that include anti-inflammatory, antioxidant, anti-proliferative, and anti-thrombotic actions [1, 2].

Heme oxygenase (HO)-1 is the inducible isoform of heme oxygenase responsible for the oxidative degradation of heme. Its products contribute to the antioxidant, anti-inflammatory and anti-apoptotic actions of HO-1 [3]. HO-1 is induced by pro and anti-inflammatory cytokines [4], lipopolysaccharide (LPS) [5] and nitric oxide (NO) [6, 7]. HO-1 has been described *in vivo* as a downstream effector of interleukin (IL)-10 [8] and to play a role in the resolution of inflammation [9].

As part of the feedback mechanisms, macrophages with anti-inflammatory activities are activated. Subsets of anti-inflammatory macrophages are characterized with the expression of arginase-1, mannose receptor-1 or the lectin C-type lectin domain family 7 member A (CLEC7A) and are referred to as Th2 –driven macrophage or M2 macrophages [10–13] important in the tissue repair and the resolution of inflammation. Multiple studies suggested a role of HO-1 induction in the polarization of macrophages into an anti-inflammatory M2 phenotype [14, 15]. Zhang et al have shown that deletion of HO-1 in the myeloid lineage exacerbates the pro-inflammatory phenotype of bone marrow-derived macrophages in response to lipopolysaccharide and limits the anti-inflammatory phenotype in response to interleukin-4 [15].

Recent studies have shown that statin induces HO-1 in murine macrophage cell lines RAW 264.7 and J774A.1, in NIH 3T3 fibroblasts and in primary murine peritoneal macrophages [16–20]. On the other hand, statins reduced the LPS-induced prostaglandin E_2_ synthesis, and cyclooxygenase-2 (COX-2) expression in monocytes [21]. However, little is known about the effect of statins *in vivo* and the mechanisms underlying its beneficial effects in inflammation [22, 23]. Statin administration to mice was shown to increase the expression of HO-1 in heart and lung tissue [24]. Few studies investigated the mechanisms involved in the role of statins in inflammation *in vivo* but did not assess the role of HO-1 [22–24]. HO-1 has been shown to play a role in the anti-inflammatory effects of some drugs including the cannabinoid receptor 2 agonist JWH-133 [25].

In the present study, we employed the air pouch model in C57BL/6 mice to assess the effect of atorvastatin on inflammation. We first determined the expression of the anti-inflammatory genes in response to atorvastatin alone and characterized the subtypes of immune cells recruited in response to zymosan and /or atorvastatin. We next demonstrated that the effect of atorvastatin on zymosan-induced leukocytes recruitment and inflammation involves HO-1 as a potential anti-inflammatory player.

## Materials and methods

### Materials

BSA, DMSO and zymosan A from Saccharomyces cerevisiae (Z4250) were from Sigma-Aldrich (St Louis, MO, USA). Tin protoporphyrin IX (SnPPIX) (Sn749-9) was obtained from Frontier Scientific (Logan, UT, USA). Atorvastatin (10493) and prostaglandin (PG) E_2_ EIA measurement reagents were from Cayman Chemicals Company (Ann Arbor, MI, USA). Kits for ELISA for mouse IL-1α (88-5019-77) and monocyte chemoattractant protein-1 (MCP-1) (88-7391-86) were purchased from Thermo Fisher Scientific (Waltham, MA USA). Antibodies for flow cytometry were from BioLegend (San Francisco, CA, USA).

### Methods

#### Subcutaneous dorsal air pouch model

C57BL/6J female mice (20–25 g, 8 week-old) were obtained from Charles River (Ecully, France) and the animal facility of the American University of Beirut. They were housed 5 per cage with cotton cocoon as enrichment environment in temperature- and humidity-controlled rooms, kept on a 12-hr light-dark cycle, and provided with food and water ad lib in the animal facility of the American University of Beirut. Body weight and food intake were monitored three times a week throughout the study period. Approval for use of animals was obtained from the Institutional Animal Care and Use Committee of the American University of Beirut (IACUC # 16-11-393).

**Fig 1:**
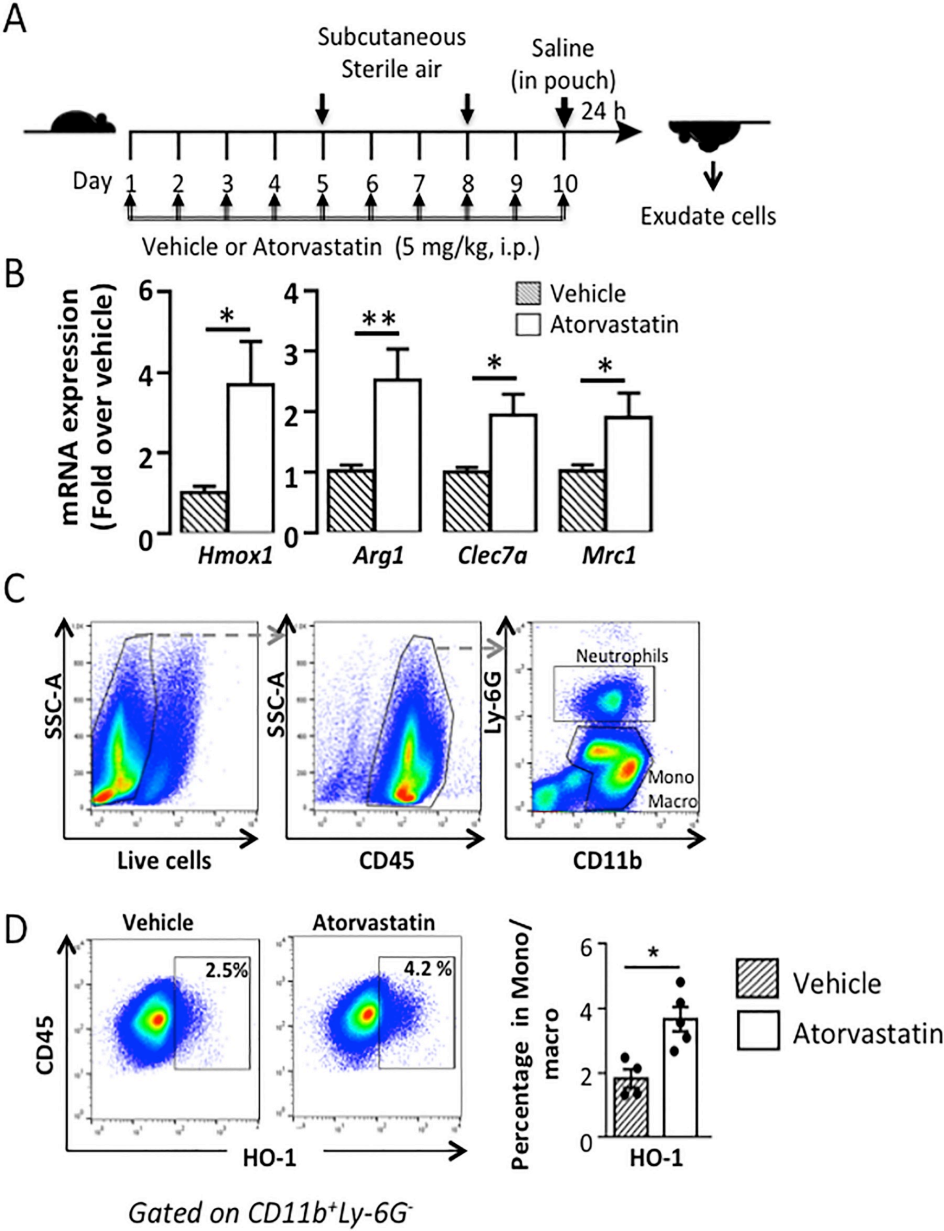
Atorvastatin induces the expression of anti-inflammatory genes in air pouch of C57BL/6J mice. A) Outline of the air pouch model. Atorvastatin (5 mg/kg, i.p.) or vehicle was injected every day for 10 days in C57BL/6J mice and cell were harvested as described in the method section, B) Gene expression of *Hmox1*, *Arg1*, *Clec7a*, and *Mrc1*. C) Representative gating strategy for the quantification of the proportion of cells expressing HO-1 cells in air pouches of vehicle- or atorvastatin-treated mice. Viable CD45^+^ cells were gated in the total exudate cells. Neutrophils were identified as viable CD45^+^CD11b^+^Ly-6G^+^ cells and were excluded from subsequent monocyte/macrophage gating. Monocyte/macrophage were selected as viable CD45^+^CD11b^+^Ly-6G^-^ cells, D) Representative flow cytometry dot plots of HO-1 expression and summary data. Mean ± SEM (n = 6). *P<0.05, **P<0.01.

**Fig 2:**
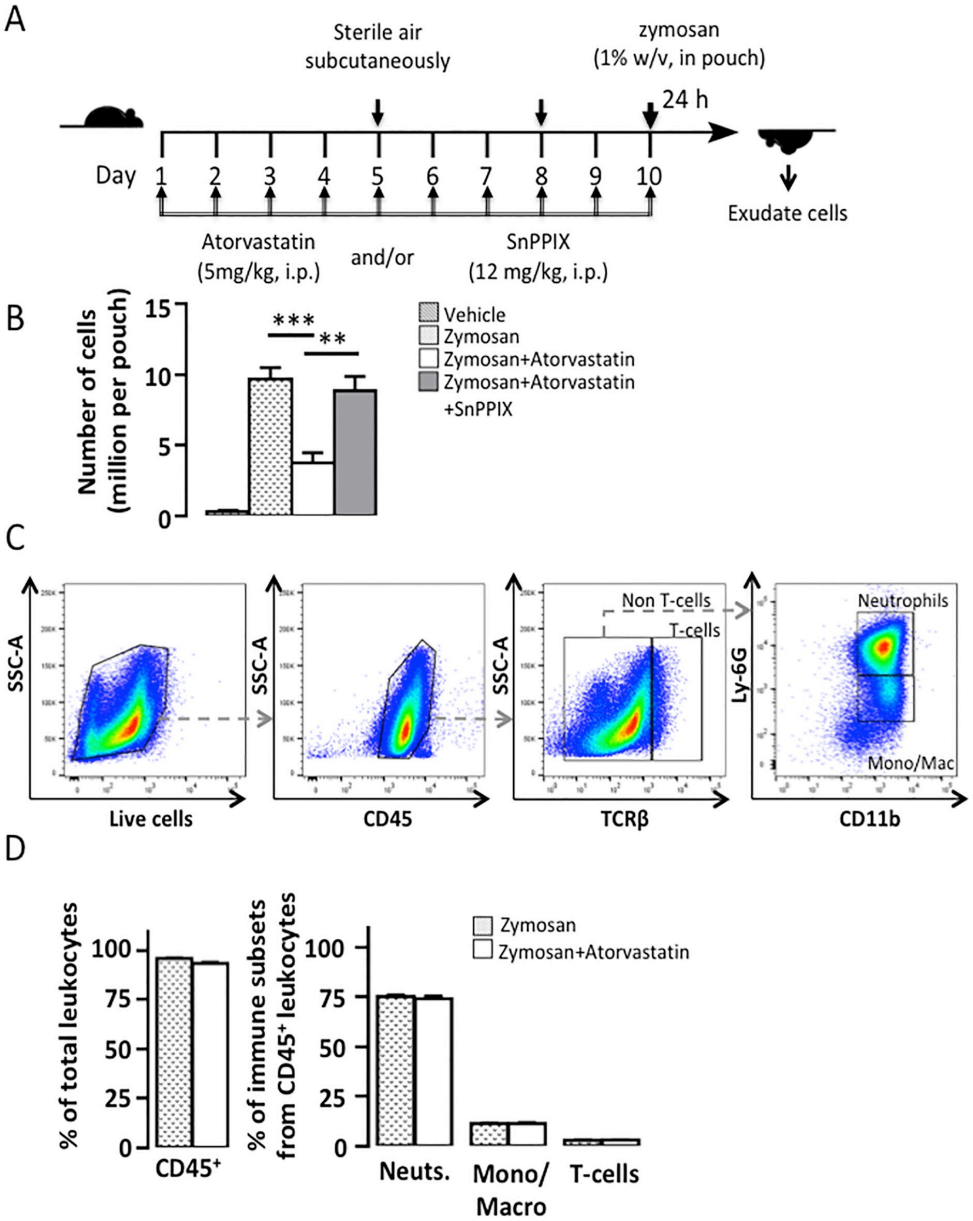
Atorvastatin inhibition of zymosan-induced cell recruitment to the air pouch is HO-1 dependent and does not involve modification of leukocyte subsets. A) Outline of the air pouch model of inflammation induced by zymosan. Atorvastatin (5 mg/kg, i.p.) and/or SnPPIX (12 mg/kg, i.p.) were injected daily for 10 days. On day 10, air pouches of mice were injected with 0.5 ml of saline and/or 1% (w/v) zymosan in saline. The exudates were collected after 24 hours. B) Number of cells the air pouches. C) Representative gating strategy for the characterization of the exudate of air pouches injected with zymosan in mice treated with vehicle or atorvastatin. Viable CD45^+^ cells were gated in the total exudate cells. T-cells were identified as viable CD45^+^TCRβ^+^ cells and were excluded form subsequent gating. Neutrophils were identified as viable CD45^+^TCRβ^-^CD11b^+^Ly-6G^+^ cells and monocyte/macrophage as viable CD45^+^TCRβ^-^CD11b^+^Ly-6G^-^ cells. D) Summary data. Mean ± SEM (n = 8–9). **p<0.01, *** p <0.001.

Atorvastatin (5 mg/kg, i.p.) was diluted in DMSO: saline, 1:49 (v:v), and SnPPIX (12 mg/kg, i.p.) in saline [26, 27] and mice were injected every day for 10 days (Figs 1A and 2A). Air pouches were established in mice as described previously [28]. Briefly, mice were anesthetized using isoflurane inhalation and air pouches were produced on day 5 by subcutaneously injecting 5 ml of sterile air into the back of the mice. On day 8, pouches were maintained by re-inflation with 2.5 ml of sterile air. On day 10, 0.5 ml of sterile saline solution or 0.5 ml of 1% zymosan in saline (w:v) was injected in the air pouch. 24 hours after the injection of zymosan, mice were sacrificed by CO_2_ inhalation and the exudates were collected in 1 ml of Hanks buffer containing 0.32% trisodium citrate to prevent cell aggregation. The number of cells in exudates was counted using improved Neubauer hemocytometer. Supernatants were kept at -80°C for the measurement of PGE_2_, mouse IL-1α and MCP-1. Total RNA was extracted from cell pellets for real time RT-PCR. For vehicle and atorvastatin–treated alone, twelve mice were injected and cells were pooled from 3 different mice. For zymosan, zymosan + atorvastatin and zymosan + atorvastatin + SnPPIX, eight mice were used in each experimental group.

#### RT-PCR analysis

Cell pellets were suspended in QIAzol (QIAGEN, 79306) and extracted as previously described [19]. 1 μg of total RNA was reversed transcribed using High-Capacity cDNA Reverse Transcription Kit (Thermo Fisher Scientific, 4368813). RT-PCR was carried out on CFX384 cycler using ABsolute Blue QPCR Mix, SYBR Green (Thermo Fisher Scientific, AB4166B) and the primers obtained from TIB Molbiol (Berlin, Germany). Oligonucleotide sequences were according to the references [29] and [30], except for *Hmox1*, *Ptgs2*, *Pges* and *Nos2*, which were as follow: *Hmox1* (F): GGCTAAGACCGCCTTCCTGCTC; *Hmox1* (R): GCAGGGGCAGTATCTTGCACCAG; *Ptgs2* (F): AGACAGATTGCTGGCCGGGTTGCT; *Ptgs2* (R): TCAATGGAGGCCTTTGCCACTGCT; *Pges* (F): GATGGAGAGCGGCCAGGTGC; *Pges* (R): GGCAAAAGCCTTCTTCCGCAGC; *Nos2* (F): CCCTTGTGCTGTTCTCAGCCCAAC; *Nos2* (R): GGACGGGTCGATGTCACAT GCA. Gene expression was normalized to the housekeeping gene 18S rRNA.

#### Flow cytometry analysis

Flow cytometry analysis of cells collected from the air pouch was performed. To characterize the inflammatory subsets in the pouch, multi-color fluorescence cell staining was conducted using the combination of the following antibodies as indicated in S1 Table: CD45 (PerCP-Cy5.5), TCR β (FITC), CD11b (BV450/50), and Ly-6G (PE). Dead cells were excluded using zombie yellow viability kit (BioLegend 423104) or Live/Dead Fixable Blue dead stain kit (Thermo Fisher Scientific, L23105). For HO-1 and CD206 detection, air pouch cells were fixed with the Fixation Buffer (BioLegend 420801) and treated with the intracellular staining permeabilization Wash Buffer (BioLegend 421002) according the manufacturer’s instructions. Rabbit polyclonal anti-HO-1 1/200 [31, 32], rat anti-rabbit IgG–FITC (Thermo Fisher Scientific F-2765), and anti-CD206 (for MRC1, BioLegend 141705) were used. Isotype controls and a control without the primary anti-HO-1 antibody were run. Three mice were pooled for the treatment with vehicle or atorvastatin alone. Analysis was performed at the faculty of medicine core facility at the AUB using FACS Aria SORP (BD Biosciences). Data were analyzed using FlowJo (TreeStar, Ashland, Or). Neutrophils were defined as living (live/dead cell stain negative) CD45^+^TCRβ^-^CD11b^+^Ly-6G^+^. T cells were defined as living CD45^+^TCRβ^-^. Infiltrating monocytes/macrophages are defined as viable CD45^+^TCRβ^-^Ly-6G^-^CD11b^+^.

#### Statistical analysis

Statistical analysis was performed using GraphPad Prism 5 (La Jolla, CA 92037 USA). Results are presented as the mean ± SEM. The level of statistical significance was determined by Mann-Whitney and p<0.05 was considered statistically significant.

## Results

### Atorvastatin induces the expression of anti-inflammatory markers in cells isolated from the sterile dorsal air pouch

We first investigated the effect of atorvastatin alone in mice. We assessed the levels of gene expression in cells isolated from the sterile cavity of the air pouch after 10 days treatment with atorvastatin (5 mg/kg, i.p.) (Fig 1A). HO-1 was significantly increased in atorvastatin-treated mice compared to untreated mice (p<0.05) (Fig 1B). We also checked the expression of some anti-inflammatory genes. Atorvastatin significantly increased the expression of arginase-1 (Arg-1) (p<0.01), C-type lectin domain family 7 member A (CLEC7A), and Mannose receptor C-type 1 (MRC1) (p<0.05). To determine the cell types that express HO-1 following atorvastatin treatment, we analyzed their phenotype in the air pouch of mice injected with atorvastatin alone (Fig 1C and S1 Fig). Leukocyte expressing HO-1 and the anti-inflammatory marker, MRC1, (also as CD206) were increased in mice treated with atorvastatin compared to vehicle. Moreover, monocyte/macrophage gated on leukocytes (CD45^+^) and expressing HO-1 were increased compared to mice treated with vehicle alone (Fig 1D).

Thus, atorvastatin significantly induced the expression of HO-1 and other anti-inflammatory markers in resident cells of the air pouch.

### HO-1 mediates the inhibitory effect of atorvastatin on zymosan-dependent cell migration

We next investigated whether HO-1 is involved in the inhibitory effect of atorvastatin on the recruitment of cells in the air pouch. Mice were treated with atorvastatin and/or SnPPIX, an inhibitor of heme oxygenase, daily for 10 days prior to inducing inflammation in the air pouch with zymosan (Fig 2A). Fig 2B shows that zymosan injection in the air pouch increased significantly the number of recruited cells compared to vehicle. Atorvastatin administration significantly reduced zymosan-induced cell recruitment by 61% (p<0.001, atorvastatin+zymosan *vs* zymosan) in response to zymosan alone. Co-treatment with SnPPIX abolished the inhibitory effect of atorvastatin on cell recruitment (p<0.01, atorvastatin+zymosan+SnPPIX *vs* atorvastatin+zymosan) indicating a role for HO-1 in the anti-chemotactic effect of atorvastatin (Fig 2B).

We further characterized the inflammatory subsets in the pouch using flow cytometry analysis (Fig 2C). Zymosan-recruited leukocytes (CD45^+^) were 96% of total viable cells in the air pouch, and consisted mainly of neutrophils (75% of CD45^+^), monocytes/macrophages (11% of CD45), and T cells (2.9% of CD45^+^). The proportions of zymosan-recruited leukocytes sub-populations were not modified by atorvastatin (Fig 2D).

### HO-1 is involved in the effect of atorvastatin on zymosan-induced expression of proinflammatory genes

**Fig 3:**
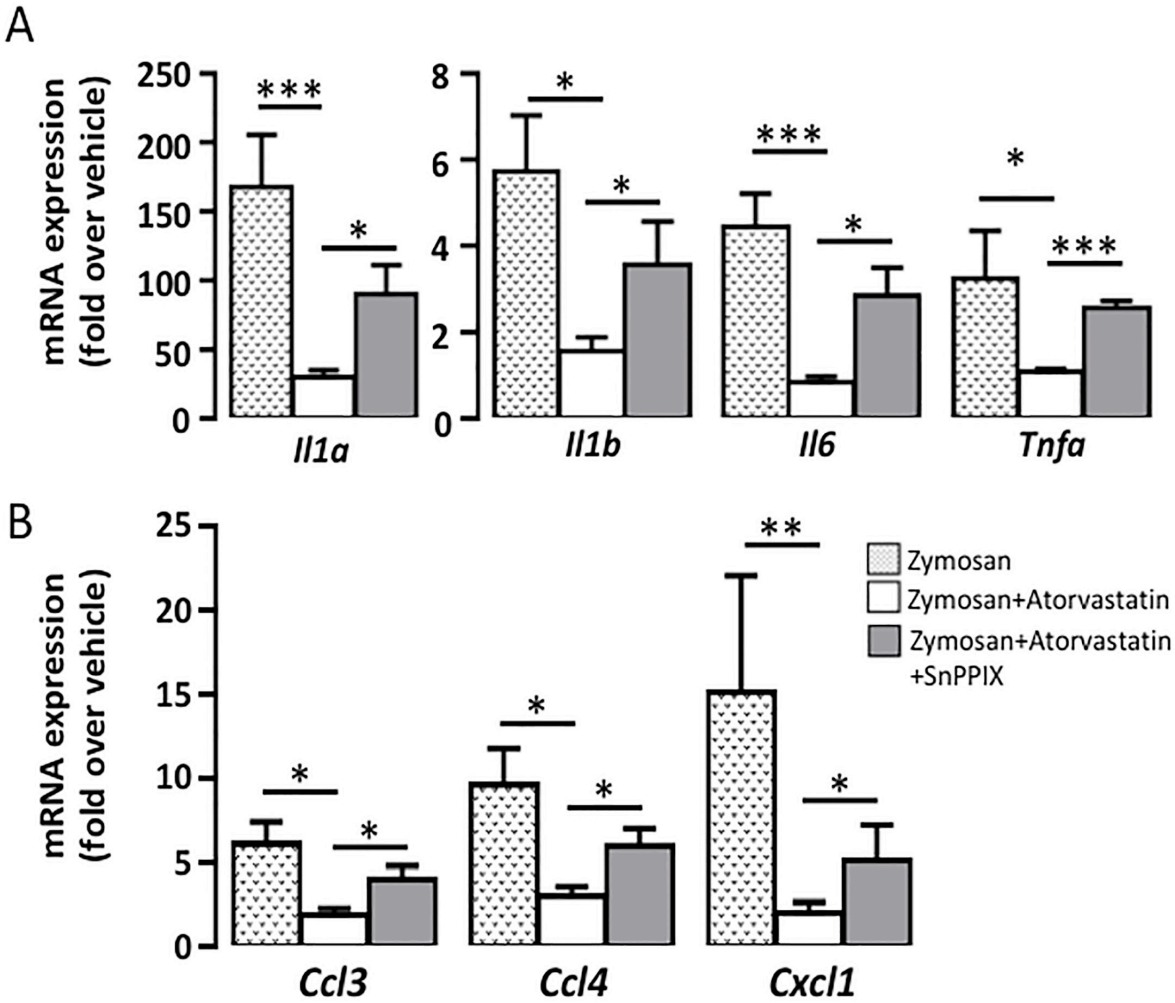
HO-1 -dependent suppression of proinflammatory cytokines and chemokines by atorvastatin. Mice were treated as described in the legend for Fig 2. Gene expression of A) Cytokines, B) Chemokines. Mean ± SEM (n = 8); * p<0.05; ** p<0.01; *** p <0.001.

Next, we analyzed the expression of some proinflammatory cytokines and chemokines. Zymosan-injected air pouches showed a significant increase in gene expression of *Il1a*, *Il1b*, *Il6*, and *Tnfa*. Atorvastatin significantly decreased the gene expression of *Il1a by 82%* (p<0.001), 73% for *Il1b by 73%* (p<0.05), *Il6 by 81%* (p<0.001), and *Tnfa by 67%* (p<0.05). Mice co-treated with SnPPIX reversed the effect of atorvastatin (Fig 3A). Fig 3B illustrates the gene expression of chemokines under the same experimental conditions. Atorvastatin also reduced the expression of *Ccl3* by 68% (p<0.05), *Ccl4* by 68% (p<0.05) and chemoattractant chemokine *Cxcl1* by 78% (p<0.01).

**Fig 4:**
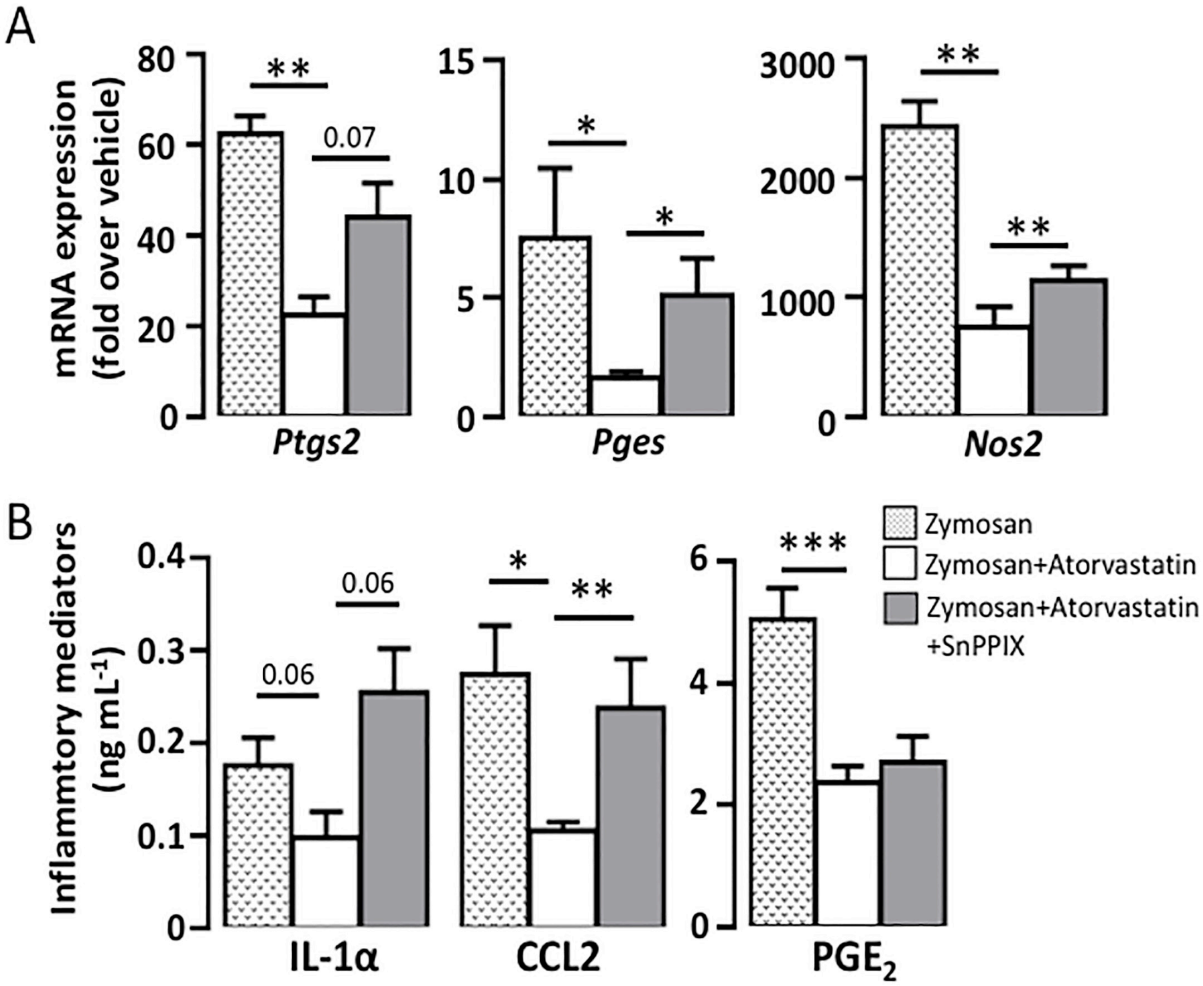
Atorvastatin- mediated inhibition of *Ptgs2*, *Pges* and *Nos2* gene expression is HO-1 dependent. Mice were treated as described in the legend for Fig 2. A) Gene expression of *Ptgs2*, *Pges*, and *Nos2*, B) IL-1**α**, MCP-1 and PGE_2_ concentration. Mean ± SEM (n = 8).

This inhibitory effect of atorvastatin on zymosan was reduced by SnPPIX treatment. Since both COX-2/mPGES-1 and NOS-II are responsible for the synthesis of proinflammatory mediators such as PGE_2_ and nitric oxide, respectively, and are important players in the inflammatory response and cytokine synthesis, and in agreement with *in vitro* statin-mediated modulation of their expression in leukocytes, we analyzed their expression in response to zymosan *in vivo*. Fig 4A shows a strong increase in gene expression of *Ptgs2*, *Pges* and *Nos2* by zymosan. Atorvastatin significantly inhibited *Ptgs2 by 64%* (p<0.01), *Pges by 83%* (p<0.05) and *Nos2* by 75% (p<0.01). SnPPIX reversed this inhibitory effect of atorvastatin.

We finally assessed the effect of atorvastatin and SnPPIX on the protein synthesis of some inflammatory mediators. Zymosan-injected air pouches showed an increased secretion of cytokine IL-1α and MCP-1 (Fig 4B). In atorvastatin-treated group, IL-1α was inhibited by 44% (p = 0.06) and MCP-1 by 71% (p<0.05) (zymosan+atorvastatin *vs* zymosan). Similarly to gene expression, SnPPIX attenuated atorvastatin inhibitory effect on IL-1α and MCP-1. PGE_2_ formation was also significantly decreased by 53% in the atorvastatin-treated group compared to zymosan (p<0.001). However, SnPPIX did not show any significant reversal effect on PGE_2_ inhibition by atorvastatin, suggesting either a HO-1-independent mechanism or a direct inhibition of the cyclooxygenase by SnPPIX since cyclooxygenase is a heme binding protein and that different protoporphyrin can compete with its heme [33].

## Discussion

It has been reported that statins have many beneficial protective effects including improvement of endothelial dysfunction, antioxidant, and anti-inflammatory effects. Statins were first shown to enhance NO production in aortic endothelial cells by activating endothelial nitric oxide synthase [34] (NOS-III) and to possess an antioxidant activity by scavenging hydroxyl and peroxyl radicals *in vitro* [35]. Moreover, statins inhibited IL-6 and IL-8 mRNA and protein expressions in LPS-stimulated human bronchoepithelial cells [36]. We have previously shown that statins inhibits COX-2, a proinflammatory enzyme in monocytes in a Rac and NF-κB–dependent manner [21]. In addition statins have been shown to induce HO-1 expression and to inhibit the production of IL-6 and TNF-α in macrophages stimulated with LPS [16, 18, 37].

Few studies have attempted to address the mechanisms of the beneficial effects of statins *in vivo*. Studies have shown an improvement of endothelial dysfunction by enhancing NOS-III expression in a rat model of pulmonary hypertension and in apolipoprotein E (ApoE)–deficient mice [38, 39]. Treatment of mice with atorvastatin or rosuvastatin had an antioxidant effect in the heart through the induction of HO-1 and the production of its products, carbon monoxide (CO) and bilirubin [40]. In the present study, we provide the *in vivo* evidence for the protective anti-inflammatory effects of atorvastatin. Our findings demonstrate that daily administration of atorvastatin for 10 days increased the gene expression of anti-inflammatory markers such as CLEC7A, Arg-1, MRC1, and HO-1 in the cells isolated from the exudate of air pouch. A significant population of the leukocyte CD45^+^cells of the cell exudate was CD45^+^CD11b^+^Ly-6G, representing mainly monocyte/macrophage/dendritic populations and expressed HO-1 in mice treated with atorvastatin.

We also demonstrated that the anti-inflammatory effect of atorvastatin involves the reduction in cell influx in the air pouch in response to zymosan injection, and this effect was abolished by treatment with the selective HO inhibitor SnPPIX. HO-1 was expressed in the leukocytes migrating into the exudates of zymosan-induced mouse air pouch in a time-dependent increase, reaching maximal expression at 24–48 h [41]. Analysis of the composition of the cells in the air pouch by flow cytometry showed a high percentage of CD45^+^ leukocytes with a predominance of neutrophils CD11b^+^Ly6G^+^ and monocytes/macrophages CD11b^+^Ly6G^-^ in response to zymosan. However, pre-treatment of air pouch with atorvastatin did not result in the modification of the percentage of any leukocyte subsets.

Interleukins and chemokines have an important role in cellular trafficking of leukocytes, and in enhancing and maintaining inflammation [42]. IL-6 production in air pouch model in mice is strongly associated with inflammation, where cellular infiltration was strongly reduced in IL-6 knockout mice [43]. In our experimental model, inhibition of the expression of these proinflammatory markers by atorvastatin was mediated via HO-1. The decrease in the inflammatory cell recruitment observed in response to atorvastatin was accompanied by a reduction in the levels of mediators measured in the air pouch. At the same time, our data showed that the modulation of the expression of the proinflammatory cytokines, chemokines and enzymes, performed on the remaining inflammatory cells in the air pouch was also significantly attenuated. These findings support an inhibitory role of atorvastatin on both the recruitment of cells in the air pouch and the regulation of gene expression. It was demonstrated that HO-1 induction resulted in reducing COX-2 and NOS-II expression and PGE_2_, nitrite, LTB_4_, IL-1β and TNF-α synthesis [41]. The role of HO-1 was further reinforced using myeloid-restricted deletion of HO-1 that revealed an increase in neutrophil infiltration and enhancement of the inflammatory mediators IL-1β, TNF-α, MMP-3, and PGE_2_, highlighting an important anti-inflammatory role of HO-1 in the zymosan-induced air pouch model [44]. Importantly, CO and biliverdin/bilirubin, the products of HO reaction, exhibit anti-inflammatory effects with a reduction of proinflammatory cytokine expression [45–49] and leukocyte–endothelial interactions, supporting a role in cell recruitments [50]. Moreover, CORM-3 and CORM-A-1, compounds that deliver CO and mimic the effect of HO-1-derived CO, have been reported to exert significant anti-inflammatory effects in addition to their cardioprotective and anti-atherogenic properties [49–51].

In line with our finding on isolated macrophages, we showed that statins inhibited the gene expression of inflammatory enzymes COX-2, NOS-II and mPGES-1 in a HO-1 dependent manner. The statin-dependent inhibition of PGE_2_ in the air pouch in mice confirmed our previous results in cultured human monocytes [21]. SnPPIX has been widely used as an HO-1 inhibitor with success [52–54] despite few HO-1 independent reports [55, 56].

## Conclusion

Our study unravels *in vivo* HO-1 as an anti-inflammatory player important in the protective effects of statins and supports both statins and HO-1 induction as promising and useful anti-inflammatory strategy *in vivo*.

## Supporting information

S1 Fig Comparison of HO-1 and MRC1 expression in CD45^+^ cells from air pouch of C57BL6/mice treated with vehicle or atorvastatin. (TIFF)

S1 Table Antibody references for flow cytometry. (DOCX)

S1 File Arrive guideline checklist. (PDF)

## Abbreviations

*Arg1*: Arginase-1 gene
*Clec7A*: C-type lectin domain family 7 member A gene
CO: carbon monoxide
COX: cyclooxygenase
*Cxcl1*: chemokine (C-X-C motif) ligand 1 gene
*Hmox1*: HO-1 gene
HO: Heme oxygenase
IL: interleukin
MCP-1: monocyte chemoattractant protein-1
*Mrc1*: Mannose receptor C-type 1 gene
NO: nitric oxide
NOS-II: NO synthase-II
PG: prostaglandin
*Ptges*: microsomal PGE synthase-1 gene
*Ptgs2*: PGH_2_ synthase-2 or COX-2
SnPPIX: Tin protoporphyrin IX
*Tnfa*: tumor necrosis factor alpha
Zym: Zymosan
