# Correction: Heme oxygenase-1—Dependent anti-inflammatory effects of atorvastatin in zymosan-injected subcutaneous air pouch in mice

**DOI:** 10.1371/journal.pone.0219773

**Published:** 2019-07-10

**Authors:** The PLOS ONE Staff

## Notice of republication

An incomplete, earlier version of this article was published in error. This article was republished on June 5, 2019 to correct for this error. The publisher apologizes for the error. Please download the article again to view the correct version.
