# Supplementary figures and images for: Heme oxygenase-1—Dependent anti-inflammatory effects of atorvastatin in zymosan-injected subcutaneous air pouch in mice
Source: PLoS One. 2019 May 9;14(5):e0216405. doi: 10.1371/journal.pone.0216405 (PMC6508873; doi:10.1371/journal.pone.0216405)

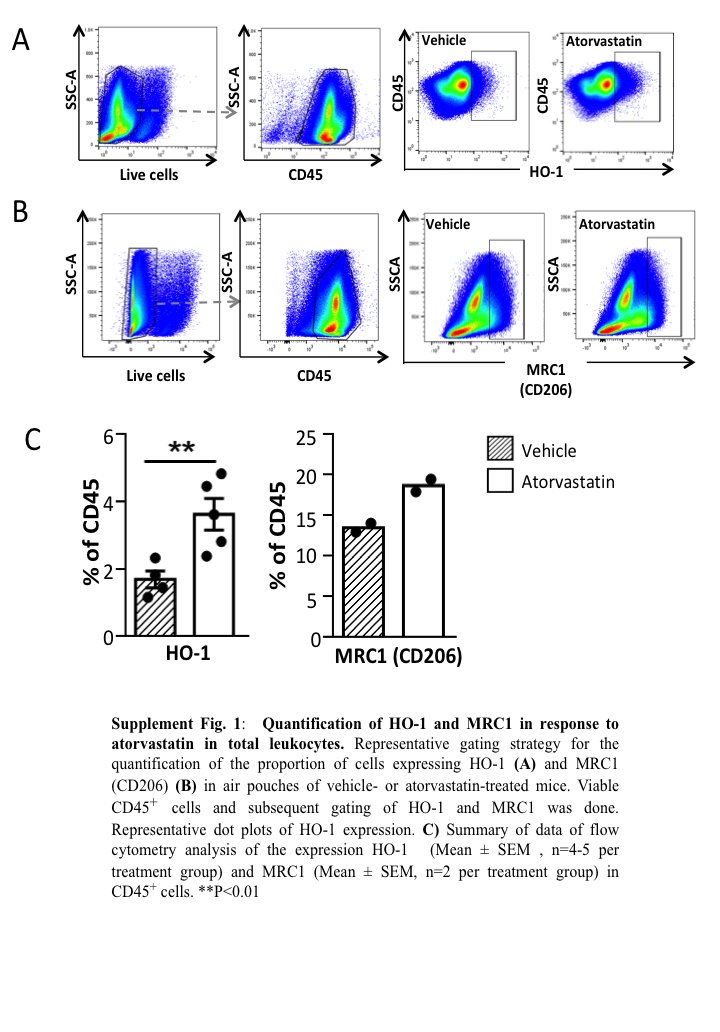

Supplement: S1 Fig — (TIFF) [file pone.0216405.s001.tiff]
