# Supplementary material for: Heme oxygenase-1—Dependent anti-inflammatory effects of atorvastatin in zymosan-injected subcutaneous air pouch in mice
Source: PLoS One. 2019 May 9;14(5):e0216405. doi: 10.1371/journal.pone.0216405 (PMC6508873; doi:10.1371/journal.pone.0216405)
Supplement: S1 Table — (DOCX) [file pone.0216405.s002.docx]

**S1 Table: Antibody references for flow cytometry.**

| **Antibody** | **Flurochrome** | **Cat. No.** | **Lot No.** |
| --- | --- | --- | --- |
| CD45 | PerCP/Cy5.5 | 103132 (Biolegend) | B218549 |
| TCRb | FITC | 109206 (Biolegend) | B202917 |
| CD11b | BV421 | 101236 (Biolegend) | B212919 |
| CD206 | BV605 | 141721(Biolegend) | B248321 |
| Ly-6G | FITC | 127605 (Biolegend) | B209826 |
| Ly-6G | PE | 127608 (Biolegend) | B221647 |
| **Isotype** | **Flurochrome** | **Cat. No.** |  |
| CD45 | PerCP/Cy5.5 | 400631 (Biolegend) | B216478 |
| TCRb | FITC | 400905 (Biolegend) | B208510 |
| CD11b | BV421 | 400639 (Biolegend) | B208409 |
| Ly6G | PE | 400507 (Biolegend) | B224315 |
